# Walking and cycling to work despite reporting an unsupportive environment: insights from a mixed-method exploration of counterintuitive findings

**DOI:** 10.1186/1471-2458-13-497

**Published:** 2013-05-24

**Authors:** Cornelia Guell, Jenna Panter, David Ogilvie

**Affiliations:** 1 Medical Research Council Epidemiology Unit and UKCRC Centre for Diet and Activity Research (CEDAR), Institute of Public Health, Cambridge, UK; 2 Present address: Faculty of Medical Sciences, University of the West Indies, Cave Hill Campus, PO Box 64, BB11000 Bridgetown, Barbados

**Keywords:** Mixed methods, Transport, Active commuting, Environmental perceptions

## Abstract

**Background:**

Perceptions of the environment appear to be associated with walking and cycling. We investigated the reasons for walking and cycling to or from work despite reporting an unsupportive route environment in a sample of commuters.

**Methods:**

This mixed-method analysis used data collected as part of the Commuting and Health in Cambridge study. 1164 participants completed questionnaires which assessed the travel modes used and time spent on the commute and the perceived environmental conditions on the route to work. A subset of 50 also completed qualitative interviews in which they discussed their experiences of commuting. Participants were included in this analysis if they reported unsupportive conditions for walking or cycling on their route (e.g. heavy traffic) in questionnaires, walked or cycled all or part of the journey to work, and completed qualitative interviews. Using content analysis of these interviews, we investigated their reasons for walking or cycling.

**Results:**

340 participants reported walking or cycling on the journey to work despite unsupportive conditions, of whom 15 also completed qualitative interviews. From these, three potential explanations emerged. First, some commuters found strategies for coping with unsupportive conditions. Participants described knowledge of the locality and opportunities for alternative routes more conducive to active commuting, as well as their cycling experience and acquired confidence to cycle in heavy traffic. Second, some commuters had other reasons for being reliant on or preferring active commuting despite adverse environments, such as childcare arrangements, enjoyment, having more control over their journey time, employers’ restrictions on car parking, or the cost of petrol or parking. Finally, some survey respondents appeared to have reported not their own environmental perceptions but those of others such as family members or ‘the public’, partly to make a political statement regarding the adversity of active commuting in their setting.

**Conclusions:**

Participants report walking and cycling to work despite adverse environmental conditions. Understanding this resilience might be just as important as investigating ‘barriers’ to cycling. These findings suggest that developing knowledge of safe walking and cycling routes, improving cycling confidence and restricting workplace parking may help to encourage walking and cycling to and from work.

## Background

Encouraging walking and cycling for transport has been suggested as one way of promoting physical activity which also confers wider public health and environmental benefits. Walking or cycling all or part of the way to work (‘active commuting’) is associated with improved health outcomes including reductions in overweight and obesity [1] and mortality risk [2,3] and greater cardio-respiratory fitness [4], while a modal shift from car use to walking and cycling for short trips would reduce carbon emissions and other adverse effects of motor vehicle use [5].

A clear understanding of the influences on walking and cycling is needed to develop interventions to encourage these behaviours. It is increasingly recognised that the environmental setting in which behaviour is undertaken might be an important influence on the behaviour itself, and this is reflected in conceptual models [6]. However, the findings of quantitative research on the environmental correlates of walking and cycling for transport are mixed, with different studies reporting positive, negative and null associations [7-9]. Some of the reported associations appear counterintuitive. For example, Titze and colleagues [8] reported that those students who perceived that the route between home and university was very safe were less likely to cycle regularly than those who reported it was unsafe. The authors suggested that respondents who cycled regularly were more likely to be aware of the danger posed by traffic than non-cyclists.

Most quantitative studies reporting these counterintuitive findings have been cross-sectional, and few qualitative studies have explored related factors that might reflect the ‘real’ reason for choosing a particular travel mode (e.g. that it is the quickest, most practical or most convenient). In addition, while many authors have explored the reasons for and barriers to walking and cycling [for example [10,11] few studies have explored the reasons why some people walk or cycle despite also reporting unsupportive conditions for those behaviours. These people are responding positively to an adverse environment and may therefore be seen as ‘resilient’, whereby resilience is conceptualised as the “dynamic process encompassing positive adaptation within the context of significant adversity” [12]. As other authors have argued, these groups may be particularly valuable to study in public health because identifying the factors underlying this resilience may help to inform interventions [13]; for example, one study in Melbourne, Australia focuses on women and children who are active and eat healthily despite living in relatively deprived areas [14]. When applied to walking and cycling, the term resilience may be used to describe those who walk or cycle despite reported exposure to circumstances that are generally regarded as more conducive to car use than to walking or cycling.

Few studies in this field have combined qualitative and quantitative data in an effort to understand engagement in walking and cycling and probe more deeply into the context of these behaviours [11,15,16]. Mixed-method research, however, is gaining increased popularity in health research and the lack of standard practice regarding how to achieve a truly mixed methodology has encouraged a lively academic debate [17,18]. The benefits of mixed-method research are seen to lie in its potential to produce complementary and therefore more comprehensive findings, and to investigate both the breadth and depth of a research problem [19]. There are various approaches to combining quantitative and qualitative datasets in meaningful ways which may be operationalised at the stages of research design, data collection or analysis. Mixed-method analysis, for example, can ‘follow a thread’ (a particular finding or variable) or ‘follow a participant’ through multiple datasets [20].

Quantitative analysis in the *Commuting and Health in Cambridge* study revealed a somewhat unexpected finding that participants who reported little traffic on the route to work were less likely to report walking to work [9]. In this paper, we aim to use qualitative data gathered about a subsample of these participants to investigate the reasons for such apparently counterintuitive quantitative associations.

## Methods

### Main study design and setting

The overall study design and recruitment procedures for the *Commuting and Health in Cambridge* study have been described elsewhere [21]. Briefly, adults working in Cambridge and living within approximately 30 km of the city centre were invited to participate through a predominantly workplace-based recruitment strategy. Participants completed a postal questionnaire that asked about travel to and from work in the last seven days, psychological measures relating to car use and perceptions of the environment on the route between home and work, as well as a range of other individual and socio-demographic factors [9]. Participants could also opt into a number of other components of the study including in-depth interviews. Quantitative data included in these analyses were collected between May and November 2009 and qualitative data were collected between June 2009 and September 2010. All participants provided written informed consent and the Hertfordshire Research Ethics Committee granted ethical approval (reference numbers 08/H0311/208 and 09/H0311/116).

### Quantitative study component

Travel modes used on the journey to and from work in the last seven days were assessed using a one-page instrument adapted from one used previously and shown to have acceptable test-retest reliability [22]. Two questions were included to assess (i) whether participants ever travelled by bicycle part or all of the way to work and (ii) the typical duration of the cycling stage of the journey (in minutes). Two analogous questions were asked for walking. From these responses, the total times spent travelling to and from work by bicycle and on foot in the last seven days were calculated and were used to identify individuals who reported any walking or cycling on the journey in the last seven days.

As travel to and from work was the primary behaviour of interest in this study, participants were asked to report their level of agreement with seven statements that could be used to describe the environment along their route to and from work using a five-point Likert scale (Table 1).

**Table 1:** Participants from the main study who reported at least three negative perceptions of the route to work and reported some walking or cycling to work in the questionnaire

	**Participants (n=340) n (%)**
Individual and household characteristics	
Age	
Under 30 years	57 (16.7)
30-40 years	93 (27.4)
40-50 years	95 (27.9)
50-60 years	65 (19.1)
Over 60 years	30 (8.9)
Weight status	
Normal weight	223 (65.9)
Overweight or obese	115 (34.1)
Gender	
Female	225 (66.2)
Male	115 (33.8)
Car access	
No car access	45 (13.2)
Car access	295 (86.8)
Driving licence	
No	29 (8.6)
Yes	310 (91.4)
Education	
Less than degree	81 (23.9)
Degree or higher	258 (76.1)
Children in the household	
No children	140 (41.2)
One or more children	200 (58.8)
Housing tenure	
Does not own their home	85 (25.0)
Owns their home	254 (75.0)
Environmental perceptions of the route	
Disagree or strongly disagree that ….	
It is pleasant to walk	84 (24.7)
There is convenient public transport	210 (61.7)
There are convenient routes for cycling	149 (43.8)
There is little traffic	442 (93.8)
It is safe to cross the road	122 (35.8)
Agree or strongly agree that…	
The roads are dangerous for cyclists	282 (82.9)
There are no convenient routes for walking	157 (46.1)

### Qualitative study component

A subsample of 50 members of the main study cohort purposively selected to provide a diverse sample in terms of age, gender and place of residence took part in a qualitative interview study in 2009 and 2010 [described in greater detail in [23]. The participants completed semi-structured interviews designed to explore their attitudes, experiences and practices and to understand how environmental and social factors interact to influence travel behaviour. Two researchers followed the same interview guide focused on travel to and from work, inquiring about typical journeys, routes, modes of transport, time and other factors (such as the need to take children to school) shaping their commuting choices and possible alternatives. The qualitative interviews did not specifically ask about environmental perceptions of the route to work and did not make reference to the participants’ questionnaire responses. Nineteen participants (plus three of their children) also took part in photo-elicitation interviews that encouraged them to explain their commuting experiences without structured questions but with the help of photographs they had produced themselves of their commuting journeys [24]. Interviews were conducted at participants’ homes or workplaces or at the research unit (according to participants’ preferences) and lasted between 20 and 60 minutes. They were audio-recorded and transcribed verbatim and the transcripts were double-checked by the researchers. Semi-structured and photo-elicitation interviews were pooled for the purposes of analysis. NVivo 8 (QSR International) was used to facilitate data management.

### Mixed-method analysis

**Figure 1:**
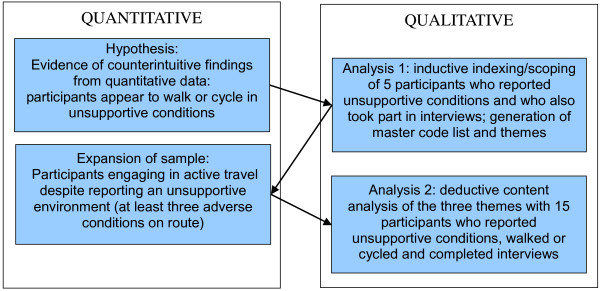
Synthesis of data.

The mixed-method analysis used an iterative, stepwise approach (Figure 1). ‘Analysis 1’ served as a scoping exercise and aimed to use the qualitative data to inductively generate codes and categories through indexing, which were synthesised into three main themes to prepare for a more systematic qualitative deductive content analysis. Five participants were initially selected for analysis on the grounds that they had given specified responses related to two environmental perceptions included in the questionnaire — they strongly agreed that there was heavy traffic on the route and that the roads along the route were dangerous for cycling — and had completed an in-depth qualitative interview. In this way, we used an adapted version of the ‘following the thread’ technique, ‘following’ individual participants and cross-referencing their qualitative and quantitative data [as others have done; [25] and comparing their experiences of journeys in these apparently unsupportive environments.

In ‘Analysis 2’, the sample was expanded to include ten further participants who had given negative responses (i.e. had strongly disagreed or disagreed with positive statements, or strongly agreed or agreed with negative statements) to at least three of the seven statements regarding the route to work (Table 1). In total, fifteen participants were identified who had reported unsupportive conditions on the route in these terms, who reported walking or cycling part or all of the journey to work, and who had also completed qualitative interviews (Table 2). Data were analysed using a systematic deductive content analysis of these participants’ interviews (and photo-elicitation interviews where applicable) using the master code list from ‘Analysis 1’, which was added to and updated during the process. The analysis was conducted by CG and peer-checked by JP. This content analysis further developed the three identified themes, expanding, interrelating and summarising subcategories to arrive at the final results.

**Table 2:** Contextual information for interview participants

**Pseudonym**	**Gender**	**Age**	**Travel context**
Andrew	Male	43	Cycles every day to work; lives a 12-minute cycle ride from work
Debbie	Female	61	Reports some walking; lives in a small village 20 miles from work without good public transport and mainly drives; at time of survey she used park-and-ride and so walked from bus stop; at time of interview six months later she drives
Frank	Male	61	Cycles four miles every day to work
Gordon	Male	68	Is given a lift by partner halfway to work and walks the rest of the way; used to cycle the 6.5 miles from his village to work
Greg	Male	61	Cycles 25 minutes every day to work from suburbs
Hannah	Female	23	Walks 10 minutes every day to the bus stop to catch the bus to work; lives in village 13 miles outside of Cambridge, from which the journey takes her 1½ hours
Isabel	Female	52	Cycles six miles every day to work from north of Cambridge to the south
John	Male	36	Cycles 15 minutes every day to work
Katie	Female	42	Cycles 15–20 minutes every day from the suburbs
Lucy	Female	45	Drives every day to the park-and-ride facility and then walks 30 minutes to work
Martin	Male	49	Usually drives the 15 miles to work but aims to cycle all the way twice a week
Pete	Male	41	Cycles every day to work; most direct route would be 10 miles and take him 35 minutes
Sam	Male	58	On four days a week car-shares with his wife to work; on fifth day, walks 20 minutes on a busy road to the railway station to travel the 15 miles to Cambridge and then walks a further 10 minutes to work
Sophie	Female	33	Usually cycles from the park-and-ride to work, but once a week she cycles the six miles all the way to work from her village outside Cambridge
Susanne	Female	39	Takes the bus to work; she also walks to the bus stop and still cycles between her two workplaces in Cambridge

## Results

From the main sample of 1164 participants who completed questionnaires, 570 participants reported three or more unsupportive conditions on their routes to and from work, and of these 340 also reported walking and cycling on the journey (Table 1). Among these participants, the most frequently reported adverse perceptions were that the roads were dangerous for cyclists and that there was a lack of convenient public transport. Further information about the purposive sample of 15 participants whose interviews (15 semi-structured, 8 photo-elicitation) were included in the mixed-method analysis is summarised separately to provide contextual background to the subsequent findings (Table 2). While all these participants reported some walking or cycling to or from work in the last seven days (which was the criterion for inclusion), some also regularly used other modes of transport in combination with walking or cycling (for example, public transport in combination with cycling) rather than walking or cycling the entire journey [26]. This may reflect the varied living circumstances of the participants that included long travel distances to work or childcare responsibilities. Three themes explaining active commuting despite reportedly unsupportive environments are presented.

### Coping strategies

The qualitative content analysis revealed that while some commuters perceived their cycling or walking route to work as unsupportive, they had found ways to adapt or rationalise their behaviour to deal with this adversity. Most were lifelong cyclists who had acquired the *knowledge* of their environment to avoid traffic or dangerous areas. This knowledge of their locality included all possible routes and their accessibility depending on season or time of the day.

Well, being a Cambridge lad [boy] I do know all the little snickets [shortcuts] and sideways and I tend to use those. . . . I’ll use the funny little footpaths, the roads that are shut off to traffic, anything that keeps me out of the traffic is good news and I’ll use it . . . Because I know most of the routes I can duck and dive a bit, it doesn’t speed things up but it makes it safer. As I say, I like to be kept away from the traffic. (Greg)

Cyclists in particular were aware of the dangers and the specific points, areas or junctions within the city where they could come into conflict with other road users, but used their knowledge and experience to avoid these.

Important in this negotiation of routes to work that might have been perceived as more acceptable was the existence of *opportunity* for such route choices. Safer routes were sometimes longer, but the extra time taken appeared to be worth it. While knowledge of such opportunities was important, the infrastructure also had to offer such alternatives. Active travel then incorporated a certain flexibility to allow for these choices.

It’s probably the safest [route], it may not be the actual shortest but I prefer it. (Katie)

I mean this adds another two or three minutes or more depending on, if you get stuck at the level crossing another five minutes. But this bit’s so much nicer . . . . I will take that time to avoid traffic there. (Frank)

Frank also explained how the traffic conditions varied by time of day, leading him to choose different routes:

Yes, yes [I have an off-road cycle path], but Long Road is always a bit difficult, pavements are blocked and the road is quite fast so in the day I cycle on the road but in the dark it’s not so appealing and the road […] is so rough.

Moreover, participants explained that their *experience* was very important in enabling them to manoeuvre their bikes through the heavy traffic or dangerous infrastructure they reported in the survey. This may also have applied to other road users.

But Cambridge drivers I think are generally aware of cyclists much better than say outsiders coming in. We get the odd one who’s a little bit impatient but there we go, that’s life. . . . It’s when you get out-of-townies who don’t really, who haven’t got a handle on cyclists at all and what they do, that’s when you get problems. (Greg)

Greg’s account of the importance of road users’ negotiation of spaces around each other suggested a tendency for car drivers in Cambridge to take account of the needs of other road users more than drivers from outside the city.

Mainly, however, such accounts referred to participants' own experience and confidence in cycling in heavy traffic or on fast roads.

Yes, that’s right, so then my strategy and all the other experienced cyclists that I know, as soon as we got into that section of the road, we would cycle out into the middle to stop anybody trying to pass us, because there really wasn’t room and we had priority. (Gordon)

You’re more aware of the fact that people might do that, so you look out for the potholes ahead of you and whether or not they’re cycling in pairs or what the situation is. You can build it into your experience. (Susanne)

Yes, well, from childhood . . . I cycled to school from when I was a teenager . . . It’s probably the most dangerous thing I do but . . . I read the statistics and it’s more dangerous not to cycle from the health point of view! *(laughs)* And I don’t believe you should give into things . . . you shouldn’t allow yourself to be forced off doing it by a whole load of selfish people in cars. (Frank)

With long-term experience of active travel, confidence could be gained through the knowledge that accidents are not as frequent as public narratives might suggest and while environments might be perceived as dangerous, everyday experience showed that these dangers could be avoided.

I think in 40-odd years of cycling I’ve only been whacked [knocked] off my bike about three times. I’ve had a few near misses but other than that … it’s not too bad. (Greg)

### Other reasons

Interviews demonstrated very clearly why some participants engaged in active commuting despite reporting their commuting route as unsupportive. There were other reasons for active travel that outweighed traffic or safety concerns or even did not allow for an alternative.

I don’t have a car and I’ve not passed my driving test, I stopped learning to drive because . . . oh I could afford a car but to have had a car and then afford to fill it up with petrol and park on site every day, well it’s just like not cost effective so I thought no, so I’ll just get the bus. (Hannah)

Other reasons included a preference for active travel over driving to gain more control over the commuting journey, in particular to save time because an active commute (including park-and-ride followed by walking or cycling) was sometimes the fastest possible way to get to work, because of heavy traffic within the congested city centre.

Because it’s quicker, I think that’s my primary reason actually. I mean if it was quicker for me to drive, if I wasn’t sitting in traffic queues then I might be tempted to drive more often. (Isabel)

It’s much nicer to be independent because you know you want to do things at different times and come home at different times. Yes, she [my wife] wouldn’t mind car sharing so much. And my patience of sitting in traffic jams for 40 minutes isn’t very good. (Frank)

Moreover, participants listed lack of parking and the cost of parking and petrol as good reasons to opt for an active commute.

Before I worked at [the hospital] I worked [in the city centre] and then they had their own big car park so I always parked up there and get there very early . . . . But the colleges don’t have any parking at all so if I got a job there that would be very difficult. (Lucy)

Exercise and health considerations were also regarded as convincing reasons for braving certain adversities inherent in cycling or walking.

I do this as a form of exercise three times a week because the sport I used to do a lot I can’t really do so much [now]. . . . I prefer the cycle route definitely. Yeah, because it just gets you out in the country, gets you sort of engaging with the environment, you can hear all birds song and even though I get tired. I’m telling myself all the time this is exercise, it’s good for my health and I’m lucky to be able to still do it. (Martin)

This also shows that active commuting was not merely reported in negative ways, in terms of trying to find coping mechanisms or lacking other alternatives. On the contrary, many participants experienced enjoyment on their way to work and this acted as a good trade-off against other adversities.

The nice thing I find about it too, it gives me that time to take off my work hat and put my home hat ongoing home and likewise coming in I can psychologically get myself ready for work. (Greg)

[I]f it was a little more dangerous I maybe wouldn’t cycle but I like cycling I mean I’ve always preferred to cycle to work rather than any other mode of transport. (Isabel)

### Public opinion and political statements

Finally, the initial inductive analysis of interview data from those who had reported unsupportive environments in their questionnaire responses found that some did not perceive traffic to be too heavy, or their routes or chosen modes of transport too dangerous, in their interviews. On the contrary, they described themselves as confident cyclists who were not concerned about adverse environmental conditions. Further exploration showed that their ‘survey opinion’ seemed to reflect a more general public discourse around road safety than their own experience. Our 15 participants in the formal content analysis did all comment on high traffic volume or dangerous routes in their interviews or photo stories, but also described many examples of reporting *environmental perceptions of others*.

There’s so many people I know that were willing to cycle, would like to cycle, but they’re scared. (Gordon)

You see I’m, what obsesses me is all the things that motorists do to me. (*Laughs*) . . . I’m not alone in that . . . everybody has experiences, yes, so when they talk about them then you find that all the cyclists will . . . get uppity very quickly because they are all . . . it’s a series of frightening experiences and yes it’s certainly put both of my daughters off. (Frank)

Participants also described their family members' negative perceptions of route safety that affected their own travel choice:

I blame the narrow cycle lanes, the rough road. And so I hurt myself quite badly then, and now my wife won’t let me cycle in town, she says it’s too dangerous, which I can’t disagree with. (Gordon)

Some participants were even keen to make a political statement about the adversity of active commuting in their setting. They stressed that ‘something needs to be done’ to encourage active travel and reduce traffic congestion.

Cambridge has been designated a cycle something city, I can’t remember the right terms, but it means we, we’re eligible for government funding. So there’s a lot of cycleway development going on in the outside villages, but . . . it’s when you get inside that it starts to fall apart, because you get situations . . . where the cycleway just disappears . . . so then you’re mixing it with all the traffic, which can be interesting. (Greg)

You can cycle on the pavement, but that’s not practical and I think it’s actually more dangerous. So this idea that people can cycle safely on the pavement where there’s hedges and cars coming out all the time . . . You should be able to cycle out here . . . It would benefit everyone, Cambridge is still a complete mess. (Gordon)

## Discussion

In this mixed-method study, we aimed to explore the motivations for walking and cycling of participants living in Cambridge, UK, who also reported unsupportive conditions for walking and cycling on their route to work in questionnaires. Although cycling in Cambridge is relatively normalised, the presence and quality of cycle infrastructure varies and has been described as “more infrastructural patchwork than a paradise” [10]. In this context, we identified three main themes based on content analysis of qualitative interviews. First, it appeared that participants had gathered knowledge and experience which enabled them to remain resilient and cope with these unsupportive conditions on their route to work. Second, it was apparent that many people endured these conditions for reasons unrelated to the environment, such as childcare commitments or car parking arrangements at work. Finally, and perhaps most interestingly, it appeared that some of the more experienced active commuters may have responded in such a way as to represent broader public opinion; it might therefore have been others, such as family members or members of the general public, who perceived cycling or walking as too dangerous and whose behaviour was affected as a result.

Understanding the motivations for walking and cycling in unsupportive environments might be just as important as investigating the barriers to cycling. All participants had reasons for enduring these adverse environmental conditions and had done so in a variety of ways — whether by acquiring experience, knowledge or confidence, by making pragmatic choices to use more convenient or cheaper travel modes or to make longer but safer journeys, or as a result of weighing up the perceived benefits and costs of the options given their own circumstances. Just as insights gained from understanding successful ‘weight maintainers’ can make novel contributions to strategies to prevent weight gain and obesity [13], we suggest that tapping into these mechanisms of resilience of active travellers who overcome their unsupportive travel environments could inform policy and practice and contribute to improved theoretical frameworks of the interaction of individual and environmental characteristics in shaping behaviour change [6,27].

We found that many of the reasons for walking or cycling in hostile environments were overcome by avoidance of certain sections of the route based on experience or knowledge. Joshi and Senior [28] conclude that existing cyclists who have experience of cycling in traffic report traffic danger as less of a threat than those who have no such experience. As such, perceptions of busyness may depend on personal circumstances, confidence or cycling ability. In this study, we also found that cyclists in particular sought to find spaces which were empty in order to physically avoid traffic and conflicts with other road users, including motor vehicles and other cyclists. It appears that building up knowledge and experience of sections of their route which were more pleasant, and using them, somehow counteracted the predominantly unsupportive and sometimes dangerous conditions which they faced on other parts of their route. In keeping with the predictions of some theories of health behaviour change [29], therefore, increasing awareness of traffic-free routes through education and promotion may improve ‘journey literacy’ about the availability of these cycle-friendly routes in the local environment. Participants also raised the issue of potential conflicts between pedestrians, cyclists and drivers of motor vehicles, but as well as trying to avoid these conflict areas, experienced cyclists also knew how to negotiate the road with other users (in particular with motorists), either to get out of their way or to be dominant in these situations. Although our findings would need to be replicated in a larger sample, they suggest that interventions to promote cycling which focus on improving cycling skills and confidence through cycle training should be continued. These views are in line with recent commitments made by the Department for Transport, which will continue to support cycle training (‘bikeability’) schemes until 2015 [5]. Despite this, Cambridge cyclists in this sample did report feeling somewhat inferior and had to yield to motorists, suggesting that even in a city known for cycling, people still report feeling unsafe in both questionnaires and interviews.

Consistent with other studies [30] and other analyses from this study [23,31,32], and with some of the predictions of conventional economic theories of travel behaviour [33], we found that commuters made the decision to walk or cycle pragmatically based on a combination of other reasons unrelated to the *route* environment, such as convenience, personal preference and both domestic and workplace constraints, including the convenience and cost of alternative modes and the availability of car parking at work. A number of these are modifiable factors and could form components of an intervention strategy to promote walking and cycling. Previous research in this sample [26,31] and elsewhere [34] has highlighted the importance of parking subsidies or charges, finding that those who have to pay for parking are less likely to use the car for commuting. Interventions focusing on restricting on-site parking at work may encourage commuters to consider making the journey on foot or by bike, while providing free or subsidised car parks off-site but within walking or cycling distance may also encourage walking and cycling for part of the journey. At the same time, we found evidence that there are factors that prevent individuals from cycling; these are fluid, vary according to people’s personal circumstances and preferences and may evolve over time [30].

Walkers and cyclists also talked about the additional benefits of walking and cycling beyond that of cost, such as enjoyment and exercise. They perceived themselves to have ‘greater control’ over journey times, were less reliant on others and enjoyed the experience, although in some cases the latter appeared to be a by-product of the barriers to alternative travel modes (such as the inconvenience of sitting in traffic or the availability of car parking). Promoting these secondary benefits may also form an important component of an intervention strategy to promote walking and cycling in some settings, particularly in congested city centres.

Finally, in interpretive qualitative analysis we also probed more deeply into less explicit explanations of the discrepancy between reported perception and behaviour. Most significantly, some interviewees — largely experienced cyclists — distinguished between their own perceptions that were more positive towards their commuting environment and the perceptions of others who did not cope as well with unsupportive environments. Other participants used their interviews as an opportunity to make a political statement that environments should be made more supportive for walking or cycling. Assuming that these interviews affirmed, contextualised and differentiated their survey responses, we suggest that their survey responses should also be read as potentially reflecting such public opinion and political statements. Indeed, a recent sociological study also conducted in Cambridge confirms the importance of cycling citizenship and cycling activism in this setting [10] and thus the possibility that survey responses might be shaped by an increasing public awareness of cycle campaigning e.g. how media discourse on nuclear power provides an essential context for interpreting survey results on nuclear power [35]. While qualitative research often includes the exploration of public discourse in its research agenda [36], survey data seem to be another potential source for such analysis.

Mixed-method research projects increasingly aim to achieve both breadth and depth to address their research problem, but there is an emergent debate as to whether such projects are simply a response to a current ‘fashion’ or reflect a true attempt to use this approach to yield new research outputs [19]. Our analysis enabled us to probe behind survey responses and find explanations and contextual data. Surveys gather data in separate sections – here, travel behaviour and environmental perceptions – without the opportunity for a participant to justify their choices. In contrast, open-ended, in-depth interviews record contextual narratives on experiences and attitudes and enable interrelations to be identified; but as qualitative inquiry does not produce representative data, the extent of coping strategies or practices of resilience should not be generalised.

The limitation of this mixed-method analysis lies in the inherent difficulty of consolidating both methods and datasets and producing a research output that allows for both qualitative and quantitative terminology. This article presents mainly qualitative findings, as our analysis aimed to explain quantitative questionnaire results with the help of contextual data from the qualitative interviews. Moreover, the study focused on the very particular setting of Cambridge and drew on a small sample of highly educated and eloquent participants who volunteered to participate in interviews. Nonetheless, as with most qualitative social research the aim of this work was to produce in-depth, exploratory and explanatory findings rather than to address expectations of representativeness or generalisability. While the integration of qualitative and quantitative methods requires careful consideration of the design and approach to analyses [37], the accumulation of ‘mixed’ bodies of evidence should be encouraged to inform a more comprehensive understanding of behaviour. For example, while the combination of qualitative interview data [32] with quantitative survey data [38] can provide greater understanding of the reporting and meaning of ‘perceptions’ of the environment, as in the current paper, this could be complemented with objective measures of behaviour such as those collected using global positioning systems (GPS) [39] to provide a more comprehensive understanding of route selection and other tactics used by cyclists to negotiate an apparently unsupportive environment. Further research should consider exploring the reasons for non-participation in walking and cycling in other settings in order to inform interventions to encourage people to choose active modes of travel in environments that are often less than ideal.

## Conclusion

This mixed-method study used qualitative interview data gathered on commuting choices to investigate and explain quantitative survey findings on the associations between choice of travel mode and environmental perceptions in the same study population. While many participants reported their route to work as unsupportive for active travel, they nonetheless cycled or walked at least parts of their commuting journey. Having explored the reasons why active travellers endured these unsupportive environments on their everyday commuting journeys, we suggest that developing commuters’ knowledge of safe walking and cycling routes, improving cycling confidence and restricting workplace parking may form part of larger strategies to encourage walking and cycling to and from work. Future research should use qualitative and quantitative data separately and in combination to confirm the findings observed here, further elucidate the reasons for behaviour and behaviour change and relate these to appropriate theories and models of behaviour change.

## Competing interests

The authors declare that they have no competing interests.

## Authors’ contributions

CG and JP conceptualised and designed the analysis. CG contributed to the qualitative data collection. CG and JP led the mixed-methods analysis. DO contributed to the conceptualisation and design of the study and the interpretation of the results. All authors were involved in drafting and revising the manuscript critically for important intellectual content and approved the final version.

## Pre-publication history

The pre-publication history for this paper can be accessed here:

http://www.biomedcentral.com/1471-2458/13/497/prepub
